# Promising proteins detected by Western blot from *Echinococcus granulosus* protoscoleces for predicting early post-surgical outcomes in CE-affected Tunisian children

**DOI:** 10.1186/s13071-021-04679-5

**Published:** 2021-03-30

**Authors:** Eya Ben Salah, Coralie Barrera, Sana Mosbahi, Bruno Gottstein, Mar Siles-Lucas, Samia Belhassen, Abdellatif Nouri, Hamouda BABBA, Laurence Millon, Wahiba Sakly

**Affiliations:** 1 Département de Biologie Clinique B, Faculté de Pharmacie, Laboratoire de Parasitologie-Mycologie Médicale Et Moléculaire, LR12ES08, Université de Monastir, 1 Rue Avicenne, 5000 Monastir, Tunisie; 2 Department of Parasitology Mycology, University Hospital of Besançon, UMR/CNRS 6249 Chrono-Environnement Research Team, University of Bourgogne- Franche-Comté, Besançon, France; 3 Pediatric Surgery Department, Fattouma Bourguiba Hospital, Medical School , Monastir, Tunisia; 4 Institute for Infectious Diseases, Faculty of Medicine, University of Bern, 3001 Bern, Switzerland; 5 Instituto de Recursos Naturales Y Agrobiología de Salamanca (IRNASA-CSIC), 37008 Salamanca, España

**Keywords:** Cystic echinococcosis, Follow-up, Western blot, Protoscolex antigens, B2t ELISA, 2B2t ELISA, Post-surgical outcome

## Abstract

**Background:**

Cystic echinococcosis (CE) affects predominantly young patients in highly endemic areas. Improved serological methods are needed for the follow-up of CE cases, especially given the high rates of post-surgical relapse that require detection as soon as possible.

**Methods:**

We designed a study to investigate the value of antigenic proteins extracted from *Echinococcus granulosus* (*E. granulosus*) protoscoleces, and of recombinant B2t and 2B2t proteins, for assessing the efficacy of surgical treatment carried out on CE-affected children. This study was performed on 278 plasma samples collected from 59 Tunisian children surgically treated for CE and monitored for 3 years post-surgery. The patients were classified according to post-surgical outcomes into a “non-relapsed” (NRCE) and a “relapsed” (RCE) group. We performed in-house ELISAs to measure anti-B2t and anti-2B2t IgG and immunoblotting for the detection of IgG against SDS-PAGE-resolved *E. granulosus* protoscoleces-specific antigens. The Wilcoxon test was applied to assess anti-B2t and anti-2B2t IgG levels. We applied the Cochran Q test to compare the distribution of immunoblotting antigenic bands between 1-month and 1-year post-surgery.

**Results:**

The probability of being “relapse-free” when a decrease in antibody titers occurred between 1 month and 1 year post-surgery was 81% and 75%, respectively, for anti-B2t and anti-2B2t IgG. We identified five protoscolex protein bands of 20, 26/27, 30, 40 and 46 kDa as highly immunoreactive by immunoblot for both RCE and NRCE patients at 1 month post-surgery, and significantly lower immunoreactivity after 1 year (*p* < 10^–4^) for NRCE compared to RCE patients. The proteins at 26/27 and 40 kDa displayed the best performance in predicting the outcome, with an 84% probability of being relapse-free when the reactivity against the 40 kDa antigen, the doublet at 26/27 kDa, or both was absent or disappeared between 1 month and 1 year post-surgery, and a 93% probability of being relapsed when both bands remained reactive or increased in intensity between the two time points.

**Conclusions:**

The B2t protein could be useful for the prediction of CE early post-surgical outcomes. The proteins of *E. granulosus* protoscoleces, especially the doublet P26/27 and P40, could be promising predictive biomarkers for the post-surgical follow-up of CE cases as well.

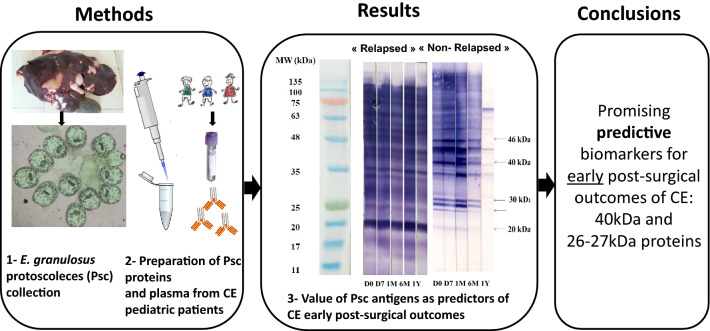

## Background

Cystic echinococcosis (CE) is a neglected tropical disease caused by the development of the *Echinococcus granulosus* (*E. granulosus*) larval stage in the human (intermediate) host [1]. Tunisia is currently one of the top endemic countries of the Mediterranean littoral [2]. CE usually remains asymptomatic for many years [3]. The incubation period and clinical manifestations depend on numerous variables, such as the cyst’s anatomic location, size, and neighboring organs [4, 5].

The diagnosis and monitoring of CE cases mainly rely on imaging techniques. Serological tests should play an ancillary, but nonetheless important, confirmatory role, particularly when imaging features are not conclusive [6, 7]. One key characteristic that marks the post-surgical follow-up period of CE is the high rate of relapses ranging from 4.6 to 22% according to different studies [8–11]. Hence, the monitoring of CE patients is mandatory for the detection of recurrences as soon as possible [12], especially in highly rural endemic areas where patients do not have easy access to imaging techniques. Over the past few years, many approaches have been described for the follow-up of CE cases including the detection of antibodies, cytokines, and parasitic antigens [6]. Nevertheless, available serological techniques lack standardization and do not provide reliable and early information about the surgical treatment efficacy; therefore, the monitoring of CE is still challenging [6, 13].

So far, the hydatid fluid (HF) was the antigenic source of reference for antibody detection in CE patients [6]. However, many studies reported that HF had relatively poor value for monitoring post-treatment developments of CE, since anti-HF immunoglobulin G (IgG) persists up to several years after effective (complete) surgical removal of the cysts, i.e. clinical cure [6, 14–16]. Thus, many researchers have attempted to find alternative antigens for better management of human CE [17]. In this regard, several recombinant proteins, mainly derived from the two most abundant and immunoreactive HF molecules, the antigen B (AgB) (AgB1, B2, B3, and B4) and antigen 5, were produced and comparatively tested [6, 18–20]. Due to its putative role in the immunopathogenesis of CE, AgB has been, so far, the most studied *E. granulosus* protein [18, 21]. It is a thermostable polymeric lipoprotein of 120–160 kDa, composed of 8 kDa subunits and is encoded by a multigenic family including at least five genetic members (AgB1–5) [21, 22]. Hernandez-Gonzalez et al. reported that B2t and 2B2t recombinant antigens derived from AgB2 (*E*. *granulosus* G1 genotype) were useful for the diagnosis and post-surgical follow-up of CE in adult patients [14, 16, 23]. Nevertheless, the authors noted that the prognostic value of these proteins was hampered by the large number of pre-operatively non-responsive CE cases [16]. Data from previous surveys showed that the peak in immune and serological responses in many cases shows up at 1 month after surgical treatment of CE [24–26]. A hypothesis was proposed that even if a pre-operative serology was negative, the patient could likely seroconvert to positive within the first month post-surgery. Hence, a comparative approach including serological results obtained at two different time points of post-surgical follow-up could provide valuable information about the outcome of surgical treatment.

In contrast to HF, only a few studies have assessed the potential usefulness of *E. granulosus* protoscolex antigens in the monitoring of human CE [26]. Veterinary medicine-based surveys have shown that many proteins of *E. granulosus* protoscoleces are immunogenic, and such antigens have been applied for the diagnosis of ovine and canine echinococcosis [26–29]. In this respect, Ben Nouir et al*.* assessed the value of protoscolex-soluble somatic antigens for the post-surgical follow-up of CE for 5 years and reported an interesting immunoreactive doublet of 27 and 28 kDa, where corresponding antibodies decreased in clinically cured patients within the first year after surgery, and strongly persisted in relapsed or non-cured cases [26]. Later, the molecular identification of this doublet led to the definition of the EgP29 protein, which was produced in a recombinant form (recP29) and described as substantially useful for the post-surgical monitoring of pediatric human CE [24]. Nonetheless, it has been reported that the value of anti-EgP29 IgG was hampered by the large number of pre-operatively non-responsive CE-confirmed patients, especially when adult. The same observance was noticed for the 2B2t ELISA test [24, 30]. Therefore, the search for novel biomarkers to evaluate CE for the early monitoring of surgical treatment outcome has become very attractive. Here, we (i) investigated by ELISA the prognostic value of B2t and 2B2t recombinant proteins to predictively evaluate the efficacy (radicality) of surgical treatment, using an approach based on the comparison of individual antibody levels between two time points of follow-up (1 month and 1 year post-surgery) of Tunisian pediatric CE patients, (ii) explored the relationship between anti-B2t, anti-2B2t IgG levels and patients’ clinical characteristics (cyst localization and treatment strategy), and (iii) assessed immunoblotting with proteins extracted from *E. granulosus* protoscoleces to identify new biomarkers that might improve the postoperative management/monitoring of human CE.

## Methods

### Study design

Our prospective study was carried out among 59 Tunisian children surgically treated for CE, aged from 3 to 16 years. Basically, patients were the same as those described in more detail in our previous investigation [31]. The diagnosis, treatment, and clinical follow-up of young patients were performed at the Pediatric Surgery Department of Fattouma Bourguiba Hospital-Monastir, Tunisia, from 2017 to 2020, during three postoperative years for each child. Depending on the thoracic or abdominal localization of cysts (or both), imaging initial diagnosis included chest radiographs, abdominal ultrasound (US), and computed tomography (CT) scans. The imaging findings allowed the classification of cysts into three categories based on the classification proposed by the World Health Organisation Informal Working Group on Echinococcosis (WHO-IWGE) with a minor modification regarding cyst activity [32, 33]: The “active” group (CE1, CE2 and CE3b stages), the “transitional” group (CE3a stage) and the “inactive” stage (CE4 and CE5). Children presenting more than one cyst were categorized according to the stage of the most active cyst. All patients underwent surgical treatment, and all cysts were removed. An adjuvant albendazole medication was given postoperatively to complicated cases (fissured, super-infected, or large cysts) and patients with multicystic disease. The duration of post-operative albendazole medication varied according to clinical presentation of patients and post-surgical development of CE (1–12 months).

### Follow-up and collection of pediatric plasma samples

A total of 278 plasma samples were collected at the time of diagnosis (D0) and subsequently at four time points which were scheduled as 1 week (D007), 1 month (D030), 6 months (D180), and 1 year (D365) post-surgery. The number of plasma specimens varied between patients, but for all cases we collected the pre-operative plasma and 1-month and 1-year post-surgery samples. Specimens were not obtained exclusively for research purposes but collected in the framework of routine and systematic post-surgical follow-up of CE. In each of the post-operative control visits, patients were monitored by means of imaging examinations (chest X-ray, abdominal or thoracic US depending on cyst localization, CT, and magnetic resonance imaging).

Pediatric CE patients were clustered into two groups based on the post-surgical clinical development. The first group included patients with no relapses detectable by means of imaging tools 3 years post-surgery (non-relapsed CE [NRCE]; *n* = 39); 18/39 NRCE patients received combined surgery and albendazole treatment. The second group consisted of cases who experienced recurrences of CE, most likely due to accidental intra-operative spillage of protoscoleces, which were detected within the first year post-surgery (relapsed CE [RCE]; *n* = 20). Only three patients of the RCE group received albendazole treatment.

Demographic and clinical data of patients are illustrated in Table 1.

**Table 1:** Overview of the clinical characteristics of the "non-relapsed" and "relapsed" cystic echinococcosis patient groups

Patients (*n*^a^)	Sex		Median age (IQR) in years	Cyst localization and type^d^								Treatment strategy	
	F	M		Lungs		Liver		Multiple^e^		Other^f^		Surgery only	Surgery plus ABZ^g^
NRCE^b^ 39	17	22	8 (4–11)	17	CE1 (13) CE2 (2) CE3a (2)	12	CE1 (8) CE2 (1) CE3a (2) CE4 (1)	8	(CE1)	2	(CE1)	21	18
RCE^c^ 20	5	15	8 (6–11)	7	CE1 (6) CE3a (1)	6	CE1	7	(CE1)	0		17	3

^a^Total number of patients

^b^NRCE. Non-relapsed cystic echinococcosis patients after 3 years post-surgery

^c^RCE. Relapsed cystic echinococcosis patients at 1 year post-surgery

^d^Cyst classification according to WHO-IWGE [34]

^e^Multiple localization means that cysts were located in the liver and the lungs (13 cases), the liver, lungs, and peritoneum (one case), and the liver, peritoneum, and brain (one case)

^f^Other locations indicate two cases: one case with a cyst detected in the spleen and one patient with a peritoneal cyst

^g^ABZ. Albendazole medication was given post-operatively to patients with complicated cases and multicystic disease as 10 mg/kg per day

### B2t and 2B2t ELISAs

We performed ELISA for the detection of total IgG against recombinant B2t and 2B2t proteins, which were produced at IRNASA-CSIC, Salamanca, Spain [14, 16, 23]. Serological testing was performed at the National Reference Center for Echinococcosis, Besançon, France, in ELISA plates coated with 0.5 µg/ml of B2t or 2B2t recombinant protein per well, as previously described [31]. Plasma samples as well as negative and positive controls (diluted 1:200) were assayed by triplicates. A horseradish peroxidase (HRP)-labeled secondary anti-IgG probe (Protein A, Cell Signaling Technology, USA) was used at 1:4000 dilution. All incubations were carried out at 37 °C for 1 h. Following triple washing, plates were incubated with the substrate for 15 min. The reaction was stopped with 1 N sulfuric acid (Sigma Aldrich, USA). The absorbance was measured at 450 nm, and results were expressed in a serological index (SI). The following formula was applied for the calculation of SI for each optical density in each plate: [P – NC]/[PC – NC]) × 100, where NC and PC stand for negative and positive controls, respectively, and P represents each patient’s plasma sample.

### Collection of protoscoleces

Sheep livers infected with larval *E. granulosus *sensu lato were examined firstly for cyst fertility then for protoscoleces viability. Parasite (G1 genotype)-infested organs were obtained from freshly slaughtered animals at the abattoir of Monastir, Tunisia. Only cysts with clear HF were included. Fertility was determined by direct microscopic observation. The viability of protoscoleces was then assessed using 0.2% eosin vital coloration and confirmed by peristaltic mobility and flame cell activity as seen under a microscope. For a better antigenic quality, only protoscoleces exhibiting up to 90% viability were retained and aseptically collected in sterile Falcon tubes. Subsequently, parasites were washed several times with sterile physiological sodium chloride solution, then subjected to three washings with PBS at pH 7.2 to eliminate floating dead protoscoleces and germinal layer remnants. Parasite pellets obtained after centrifugation at 10,000×*g* for 15 min at 4 °C were supplemented with an equal volume of sterile sodium chloride 0.3% solution and stored at −80 °C.

### Antigen preparation

Protoscoleces soluble and membrane antigens of *E. granulosus* (PSMAs) were extracted using X-100 Triton detergent as previously described by Müller et al. [35]. Briefly, frozen parasite pellets were suspended in ice-cold 0.5 ml PBS/phenylmethylsulfonyl fluoride (PMSF) 1 mM/Triton X-100 0.1% and ice-vortexed (alternating) for 5 min. The treated pellet was then sedimented at 13,000×*g* for 10 min at 4 °C. The obtained supernatant (soluble proteins) was transferred to a new tube, and extraction was repeated twice on the same pellet to obtain membrane antigens. Subsequently, the three supernatants were combined. Protein concentration was determined by measuring the absorbance at 280 nm (A280) using a NanoDrop 2000/2000c spectrophotometer (Thermo Fischer Scientific, Wilmington, DE, USA); then, supernatants were stored at −80 °C until processing.

### Sodium dodecyl sulfate-polyacrylamide gel electrophoresis (SDS-PAGE) and immunoblotting

The PSMAs were solubilized with SDS sample buffer (0.5 M Tris–HCl pH 6.8, 2% SDS, 10% glycerol, 10% β-mercaptoethanol, and 0.004% bromophenol blue) and heated for 5 min at 100 °C. The molecular weight marker (BLUeye Prestained Protein Marker, Jena Bioscience) and treated proteins (final concentration of 150 µg/gel) were then loaded into an 8–20% linear gradient polyacrylamide gel and electrophoretically separated at 150 V for 2 h. Resolved proteins were then transferred onto a 0.45-µm nitrocellulose membrane as described by Poretti et al. [36]*.* Afterwards, nitrocellulose strips were blocked with 5% skim milk in PBS at pH 7.2 containing 0.2% Tween 20 (working solution) for 1 h, then incubated overnight with human plasma diluted at 1:100 in the working solution at 4 °C. Subsequently, strips were washed, then incubated with goat anti-human IgG conjugated with alkaline phosphatase (Vector Laboratories, USA) (diluted 1:1000) for 1.5 h. After three washings, immunoreactive bands were visualized using a solution of 5-bromo-4-chloro-3-indolyl phosphate (BCIP) and nitroblue tetrazolium (NBT). Western blot (WB) profiles were analyzed for each patient after plotting the molecular weight calibration curve. Firstly, the presence or absence of the relevant immunoreactive bands was determined for each time point of the monitoring period. Afterwards, we calculated the frequency of the presence of each band for both NRCE and RCE groups for the corresponding time point using the following formula: sum of NRCE or RCE patients displaying the band of interest / sum of all patients displaying the same band regardless of clinical status. Secondly, the band intensity was empirically determined in three levels (absence, low or high intensity) and compared in samples obtained 1 month post-surgery versus specimens collected after 1 year. Then the evolution between the two time points was categorized according to the following classification: (i) WB band remained not visible or totally disappeared, (ii) WB band had lower intensity but remained visible, or (iii) WB band yielded the same or greater intensity. Based on this classification, the probabilities of “relapse-free” and “relapse” were calculated.

### Ethical considerations

We obtained written informed consent from each parent/legal guardian of children diagnosed with CE before any collection of biological samples. This study was approved by the Ethics Committee of the Faculty of Medicine, Monastir, Tunisia (Acceptance Report IORG 0009738 N°22/OMB 0990-0279). Patients' relevant medical history and clinical data were recorded and anonymously treated. Protoscoleces were collected from parasite-infested organs during the postmortem routine inspection of animal carcasses by the official veterinarian and with his due consent. We performed no experimentation on animals; therefore, no approval from the Institutional Animal Ethics Committee was needed.

### Statistical analyses

Anti-B2t and anti-2B2t IgG levels were assessed throughout the course of follow-up, using the Wilcoxon signed-rank test for paired samples in (i) NRCE versus RCE patients, (ii) the six subgroups clustered according to cyst localization [(NRCE-liver (*n* = 12), RCE-liver (*n* = 6), NRCE-lung (*n* = 17), RCE-lung (*n* = 7), NRCE-multilocalization (cysts localized in lung and liver) (*n* = 8), RCE-multilocalization (*n* = 7)], and (iii) NRCE-treated solely by surgery (*n* = 21) versus NRCE with combined albendazole therapy (*n* = 18). Anti-B2t and anti-2B2t IgG levels could not be assessed in the RCE patients treated only by surgery versus RCE patients with combined albendazole therapy because of the low number of RCE patients who were given albendazole (*n* = 3).

To investigate the value of anti-B2t and anti-2B2t IgG as early predictors of surgical treatment efficacy, the evolution of levels from 1 month to 1 year post-surgery was evaluated as an increase, a decrease, or a stability as follows: a factor of ± 2 coefficient of variation (CV)% was applied on the first time point (D030) level and compared to the second one (D365). If the second level was higher than the previous one by +2 CV%, the level was considered as increased. In the same way, if it was lower than the previous level by −2CV%, we considered it as a decrease in level, and if it was between the high and low range, it was considered stable. The global CV of the in-house ELISA for the measurement of anti-B2t and anti-2B2t IgG was estimated to range at ± 10%. Then, the chi-square test was applied to compare the number of NRCE and RCE patients presenting a decrease of anti-B2t and anti-2B2t IgG levels between D030 and D365.

We applied the Cochran Q test to compare the distribution of immunoblotting protein bands between 1 month and 1 year post-surgery.

All continuous variables were expressed as median and interquartile range (IQR), and categorical variables as number and percentage. Box plots were plotted as median and IQR. All statistical tests were two-sided, and a probability value of *p* < 0.05 was considered to be statistically significant. *P* values in graphs are shown as *p < 0.05, **p < 0.01, and ***p < 0.001. Statistical analyses of the data were performed using R (version 3.6.1) and GraphPad Prism 8. (version 3.0.1) software.

## Results

### Anti-B2t and anti-2B2t IgG levels in NRCE and RCE patients during the different time points of the monitoring period

**Fig. 1:**
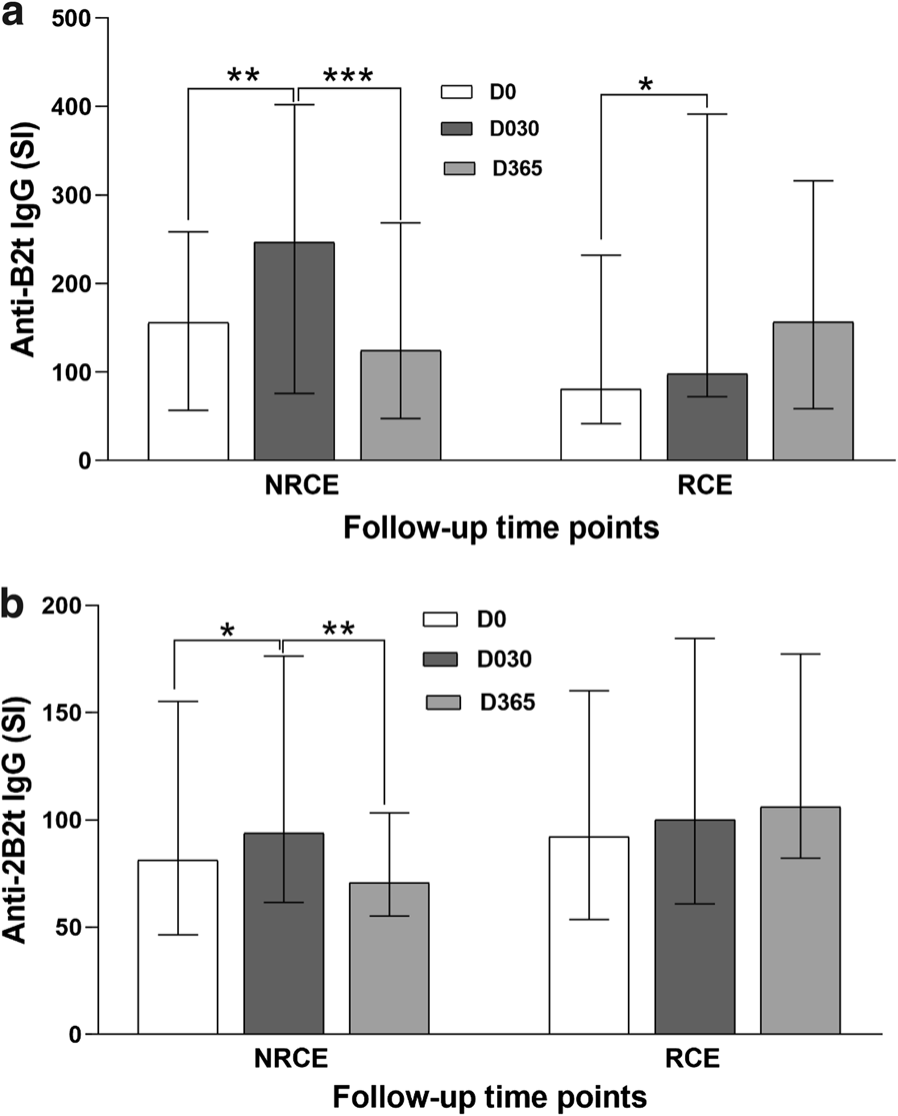
Anti-B2t (**a**) and anti-2B2t (**b**) median IgG levels (SI) in NRCE and RCE patients. NRCE, no relapsed patients. RCE, relapsed patients. D0, the time of diagnosis. D030 and D365; 1 month and 1 year post-surgery, respectively. *p < 0.05, **p < 0.01, ***p < 0.001

The evolution patterns of anti-B2t and anti-2B2t IgG were variable within each patient group **(**Fig. 1a, b**)**. In NRCE patients, the median values of anti-B2t and anti-2B2t IgG antibodies displayed the highest level at 1 month after surgery (approximately 1.6 and 1.2 times higher than pre-operative levels in terms of median values for anti-B2t and anti-2B2t IgG, respectively). Subsequently, the median values of both anti-B2t and anti-2B2t IgG were significantly lower at the end point of the monitoring period (approximately 0.5 and 0.8 times lower in terms of median values of the group for anti-B2t and anti-2B2t IgG, respectively). In contrast to NRCE, the RCE group displayed no significant variations in IgG levels between D030 and D365 against both proteins (Fig. 1a, b).

### Influence of patients’ clinical variables on anti-B2t and anti-2B2t IgG levels

**Fig. 2:**
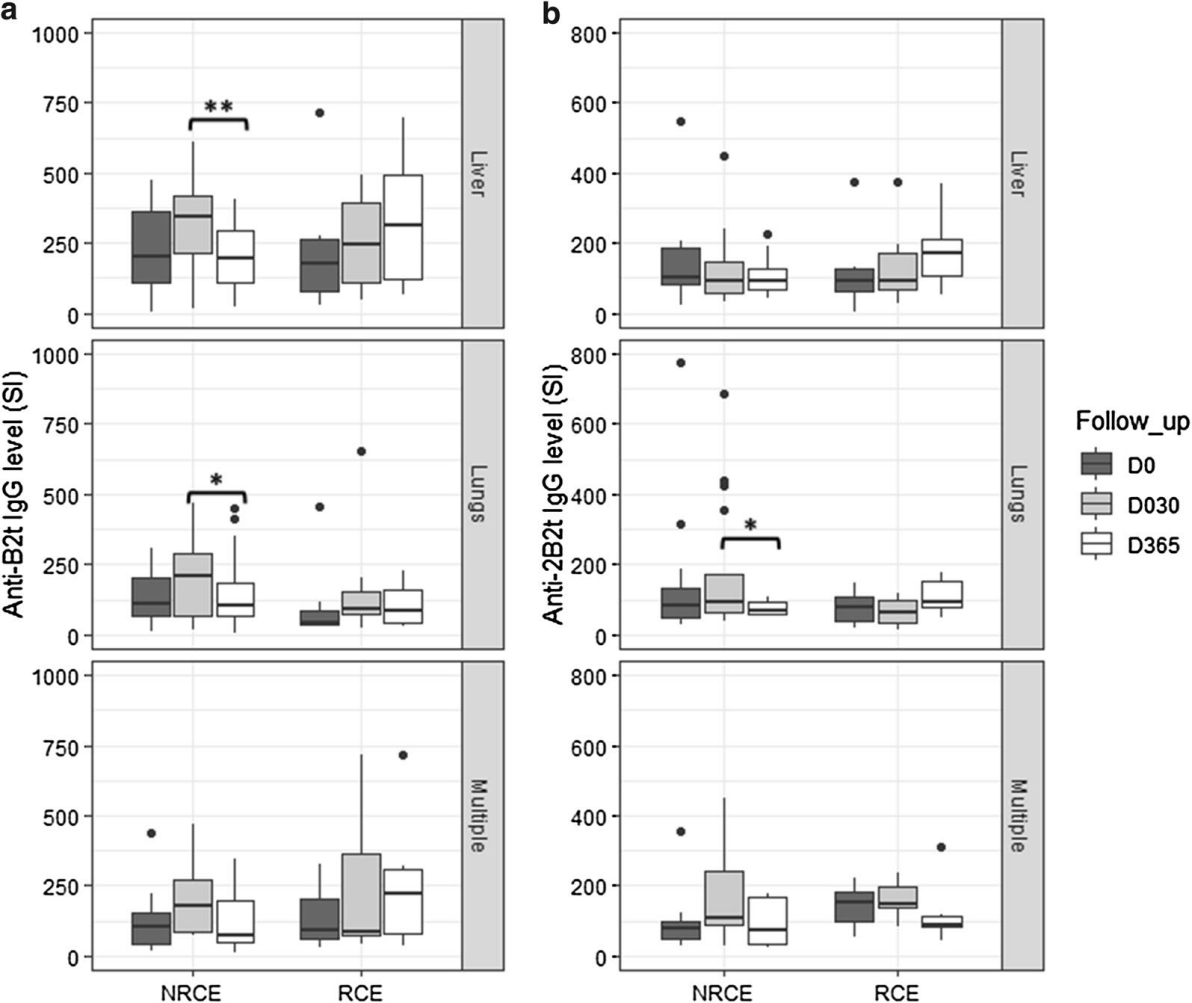
**a** Evolution of anti-B2t IgG levels according to cyst localization in NRCE and RCE. **b** Evolution of anti-2B2t IgG levels according to cyst localization in NRCE and RCE

With respect to cyst location, anti-B2t and anti-2B2t IgG levels were significantly lower at D365 than D30 in NRCE-lungs but not in NRCE-multilocalization (Fig. 2a, b). The anti-B2t IgG level was also significantly lower at D365 than D30 in NRCE-liver.

**Fig. 3:**
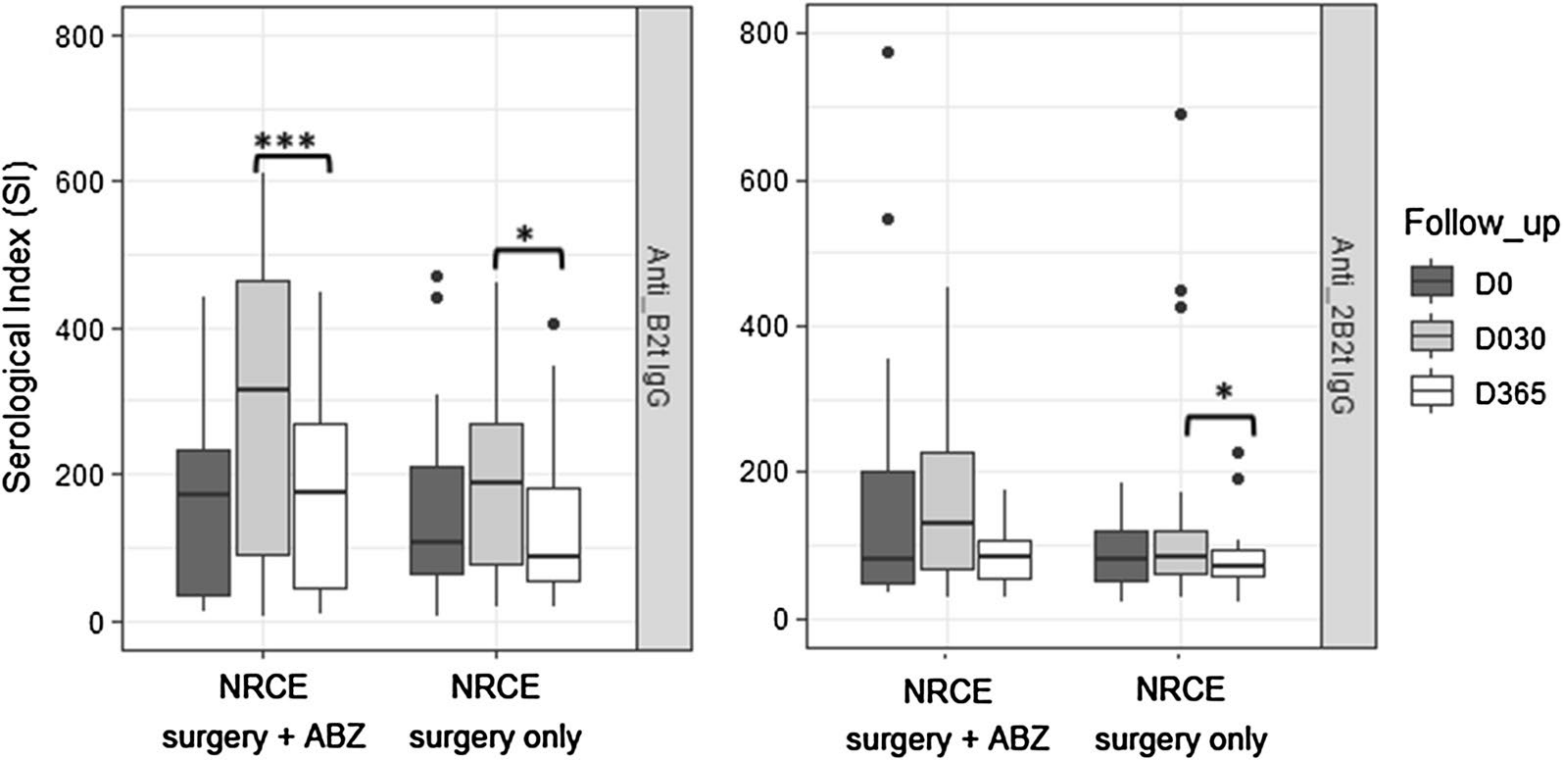
Effect of treatment strategy on anti-B2t and anti-2B2t IgG levels at different time points of follow-up period in NRCE. NRCE surgery + ABZ, NRCE with surgery and combined albendazole chemotherapy. NRCE surgery only, NRCE treated solely by surgery. *p < 0.05, ***p < 0.001

Interestingly, we noted that anti-B2t IgG levels in the NRCE group with combined albendazole therapy displayed a more marked decrease (*p* < 0.001) at 1 year post-surgery, as compared to anti-B2t levels in the NRCE group treated exclusively with surgery (*p* < 0.05) (Fig. 3). For anti-2B2t IgG level, the decrease was significant only in the NRCE group with exclusively surgical treatment (Fig. 3).

NRCE, No relapsed patients. RCE, relapsed patients. Liver: patients who have one or more cysts exclusively in the liver (12 NRCE, 6 RCE); Lungs: patients who have one or more cysts exclusively in the lungs (17 NRCE, 7 RCE); Multiple: patients who have two or more cysts in liver and lungs (8 NRCE, 7 RCE). Two patients who have cysts in other organs were not represented. * *p* < 0.05, **p < 0.01**.**

### Anti-B2t and anti-2B2t IgG antibodies as predictors for estimating early surgical treatment efficacy

Anti-B2t IgG decreased between D030 and D365 in 31 patients (25 NRCE and six RCE). Hence, the probability of being relapse-free when the level of anti-B2t IgG decreased was 81% (25/31). Anti-2B2t IgG decreased in 28 patients (21 NRCE and 7 RCE) between D030 and D365. Hence, the probability of being relapse-free when the level of anti-2B2t IgG decreased was 75% (21/28). The difference between the number of NRCE and RCE patients displaying a decreased value between D030 and D365 was statistically significant only for anti-B2t IgG (*χ*^2^ test, *p* = 0.01) (Table 2).

**Table 2:** Number of CE patients according to the evolution of IgG levels observed between D030 and D365 for anti-B2t, anti-2B2t, and both antibodies (2B2t or B2t), and determination of the probability of being relapse-free

Antibody response	Evolution	NRCE, *n* (%)	RCE, *n* (%)	Probability of being relapse-free (%)
Anti-B2t IgG	Decrease*	25 (64)	6 (30)	81
	Increase	5 (13)	8 (40)	
	Stability	9 (23)	6 (30)	
Anti-2B2t IgG	Decrease	21 (54)	7 (35)	75
	Increase	9 (23)	10 (50)	
	Stability	9 (23)	3 (15)	
Anti-(B2t or 2B2t) IgG	Decrease*	32 (82)	9 (45)	78
	Increase	4 (10)	7 (35)	
	Stability	3 (8)	4 (20)	

*NRCE* non-relapsed patients, *RCE* relapsed patients

*p < 0.05 (chi-square test)

We further analyzed the individual variation when considering a decrease between D030 and D365, if it was perceived at least for one antibody, either anti-B2t or anti-2B2t IgG. Anti-(B2t or 2B2t) IgG decreased in 41 patients (32 NRCE and nine RCE). Thus, the probability of being relapse-free was 78% (32/41) if the level decreased between D30 and D365. The difference between the number of NRCE and RCE patients displaying a decreased level between D030 and D365 was statistically significant (*χ*^2^, *p* = 0.04) (Table 2).

### Western blotting using *E. granulosus* protoscoleces proteins for monitoring CE

**Fig. 4:**
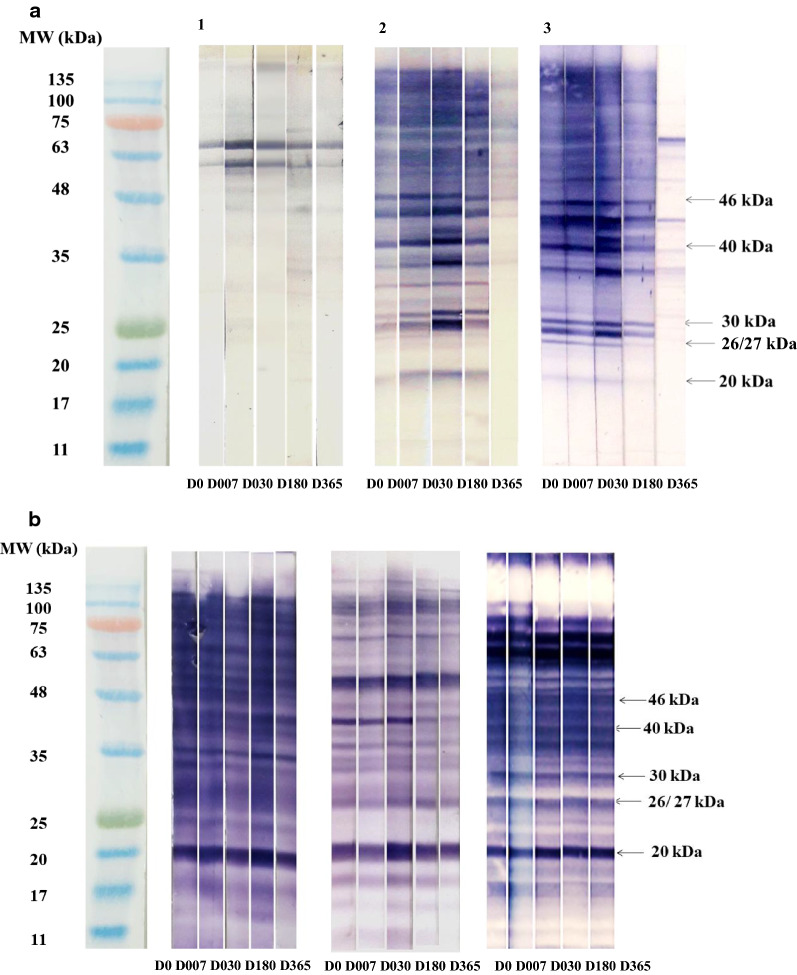
**a** Follow-up immunoblotting profiles of three “non-relapsed” (NRCE) patients. *1* NRCE with no response to the five immunoreactive bands. *2 *and 3 NRCE responsive to the five bands. **b**. Follow-up of three “relapsed” (RCE) patients. MW, molecular weight marker. D0, plasma sample collected before surgery. D007, D030, D180, and D365, plasma specimens were collected 1 week, 1 month, 6 months, and 1 year post-surgery, respectively

The PSMA immunoblotting profiles showed multiple immunoreactive bands ranging between 11 and 120 kDa. The banding pattern was heterogenous within each patient group. Interestingly however, we noticed that most CE plasma samples displayed five characteristic immunoreactive bands of molecular weights (MW) 46 (P46), 40 (P40), 30 (P30), doublet 26/27 (P26/27), and 20 (P20) kDa. Furthermore, these protein bands exhibited particular temporal fluctuations during the monitoring period. In most NRCE patients, the five antigenic bands showed nearly the same variation, marked by weakly detectable immunoreactivity at the time of diagnosis, which increased progressively to reach the highest intensity 1 month after surgery. Subsequently, antibodies against the respective antigens started to decrease gradually within 6 months after operative treatment, and corresponding bands became weakly recognizable or totally disappeared at 1 year post-surgery. By contrast, most patients of the RCE group displayed strongly intense bands throughout all the monitoring time points (Fig. 4).

Band profiles observed at 1 month post-surgery differed significantly among patients. In fact, only seven patients (12%) had a profile with the five bands, whereas most cases (29%) showed a profile with four bands. Two patients did not show any of the five bands at the time of diagnosis or during the entire follow-up period (one NRCE patient and one RCE patient); both cases had single pulmonary cysts. Considering the presence of at least one of the five immunoreactive bands at 1 month post-surgery, 38/39 (97%) and 19/20 (95%) of NRCE and RCE patients, respectively, exhibited detectable positive response to PSMAs (Table 3).

**Table 3:** Distribution of reactive bands in immunoblotting profiles of cystic echinococcosis (CE) non-relapsed (NRCE) and relapsed (RCE) patients 1 month post-surgery

Number of patients	Total (*n* = 59)	NRCE (*n* = 39)	RCE (*n* = 20)
Five protein bands (*n*, %)	7 (12)	5 (13)	2 (10)
Four protein bands (*n*, %)	17 (29)	11 (28)	6 (30)
Three protein bands (*n*, %)	16 (27)	12 (31)	4 (20)
Two protein bands (*n*, %)	11 (19)	7 (18)	4 (40)
One protein band (*n*, %)	6 (10)	3 (8)	3 (15)
Zero protein bands (*n*, %)	2 (3)	1 (3)	1 (5)
Positive response to at least one band (*n*, %)	57 (97)	38 (97)	19 (95)

*NRCE* non-relapsed cystic echinococcosis patients at 1 year post-surgery. *RCE* Relapsed cystic echinococcosis patients at 1 year post-surgery

**Fig. 5:**
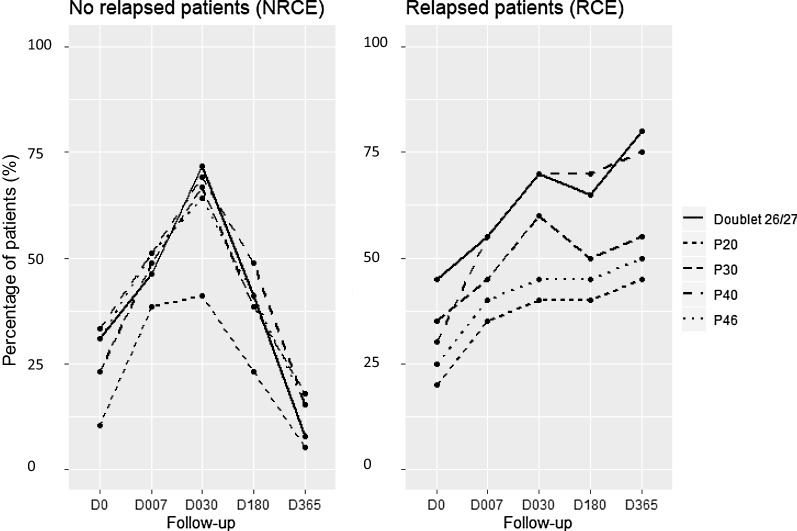
Percentage of NRCE and RCE patients with presence of a specific band at the main time points of monitoring

In the NRCE group, the five protein bands were observed less frequently at 1 year after surgery as compared to 1 month (Cochran test, *p* value < 10^–4^ for the five protein bands), in contrast to RCE patients, who exhibited unchanged frequency for the five antigenic bands between the two time points (Cochran test, *p *value > 0.5) (Fig. 5). The doublet P26/27 and P40 appear to be good biomarkers for CE, as these protein bands were the most frequent at D030 in NRCE and at D365 post-surgery in RCE (Fig. 5). Throughout the follow-up period, we observed that the frequency of occurrence for P30 and P46 was 67% and 64% for NRCE and 60% and 50% for RCE, respectively. The P20 was the least frequently detected immunoreactive band, in 41% of NRCE and 45% of RCE (Fig. 5).

**Fig. 6:**
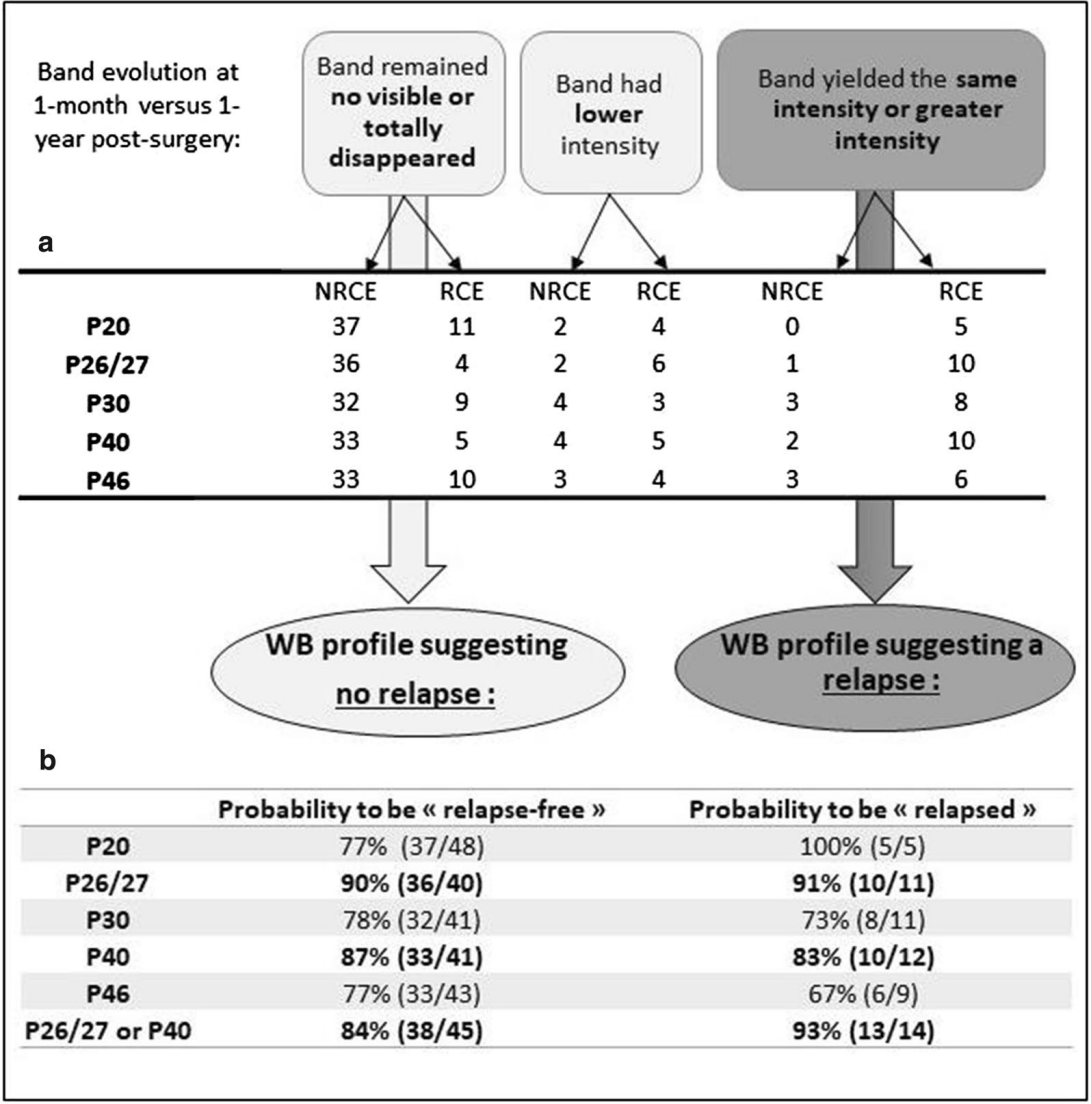
Schematic illustration of WB profile classifications according to the immunoreactivity evolution of each band at 1 month versus 1 year post-surgery. **a** Number of CE patients with the three observed WB profiles. **b** Probability of being relapse-free or relapsed according to the Western blot band evolution

According to the classification of WB profiles (Fig. 6), most of the NRCE patients had an evolution pattern that indicated a decrease of antibody response at 1 year post-surgery, while most of the RCE patients showed a stable or an increased antibody response (Fig. 6a). The probability of being relapse-free when the band remained not visible or totally disappeared was the highest for P26/27 (90%) followed by P40 (87%), while the P20 displayed the highest probability of relapse (100%) when it yielded the same or greater intensity at 1 year post-surgery (however, this persistent band was observed only in five RCE patients) (Fig. 6a, b).

A few patients showed some bands with lower intensity at 1 year post-surgery, but this criterion was subjective and was not used for the calculation of the probability of being relapse-free.

When we assessed the value of P40 and doublet P26/27 combined, as these fractions constituted the most abundant immunoreactive proteins, we found that they were detected simultaneously in 51% of patients (30 patients: 19 NRCE and 11 RCE). Considering the presence of either the P40 or doublet P26/27 or both proteins, follow-up of CE patients was achieved in 90% of patients (53 patients). Hence, the probability of being relapse-free when the P40, doublet P26/27, or both bands remained not visible or totally disappeared between D030 and D365 post-surgery was 84% (38 NRCE/45 patients). Accordingly, the probability of being relapsed when the P40, the doublet P26/27, or both bands increased in intensity or remained unchanged between D030 and D365 was 93% (13 RCE/14 patients).

## Discussion

Both B2t and 2B2t ELISA and Western blotting showed that antibody response increased considerably at 1 month post-surgery in most pediatric CE patients. Subsequently, antibody levels were lower at 1 year post-surgery for the non-relapsed group. By contrast, the relapsed group showed relatively stable antibody levels. These findings confirmed results of previous studies [14, 26]. The persistence of unchanged antibody levels in relapsed cases throughout the course of monitoring is an indication of continuous antigen stimulation and host immune response, most likely due to an accidental spillage of parasitic material during surgery or as a consequence of inadequately treated cysts in the first operation.

The analysis of individual variation in antibody levels against both recombinant proteins showed that there was an 81% probability of being relapse-free for anti-B2t IgG if a decrease occurred between 1 month and 1 year post-surgery. Performance was lower using 2B2t antigen. These findings correlate in part with data published by Gonzalez et al. (2018), who reported that the B2t ELISA was better than 2B2t ELISA in discriminating between cured and non-cured patients [16]. However, these results were only found in patients whose serology against the B2t antigen was positive already at the time of diagnosis, which actually represented a very low percentage of their study population (33%) [16]. With our comparative approach, no positivity threshold was considered, and the evaluation of post-surgical outcomes based on serological results at D030 and D365 was applicable for all patients.

Our results showed that antibody levels against both recombinant antigens were found to be lower 1 year post-surgery in non-relapsed patients with hepatic and pulmonary cysts, but not in those with multiple lesions. This observation correlates with the fact that the development of CE is closely dependent on the cysts' clinical features such as the number, stage, and anatomical localization [37].

In our study, CE patients treated solely by surgery were more likely to develop relapses in comparison to those who underwent a combined albendazole medication. Furthermore, the levels of anti-B2t IgG were remarkably lower 1 year after surgery in NRCE patients with adjuvant albendazole therapy as compared to those treated exclusively by surgery. Such observations support current knowledge that treating CE by surgical procedures jointly with chemotherapy improves the prognosis of CE and reduces the recurrence rate [38]. Unfortunately, more than half of the CE patients enrolled in the present study could not receive a combined albendazole medication, for main reasons: albendazole is a high-priced drug, and its availability is limited in our endemic area. Hence, priority access to chemotherapy was given to complicated cases and patients with multiple cysts.

Immunoblotting with PSMA revealed five characteristic immunoreactive bands of MWs 46, 40, and 30, doublet 26/27, and 20 kDa. The most detectable bands among pediatric CE patients were the doublet P26/27 (47 children) and P40 (45 cases). The disappearance of P26/27 at D365 was associated with the highest probability of being relapse-free (90%), and its persistence at the same date was associated with a 91% probability of relapse. Hence, it could be an interesting candidate for the post-surgical follow-up of CE. These findings correlate with those previously reported by our research team in 2006, where a doublet of 27/28 kDa that may be identical to the 26/27 immunoreactive double band was identified [26]. Later, the molecular identification of that doublet led to the characterization of a P29 *E. granulosus* protein that was then produced in a recombinant form [24]. Despite exhibiting a good value for monitoring CE, several authors reported that anti-P29 IgG were not detectable using ELISA in a certain portion of confirmed CE patients at the time of the diagnosis [24, 30]. Thus, if the identified doublet corresponds to the P29 protein, our findings show that it is detectable by immunoblotting in most patients, and semiquantitative measurement of specific P26/27 (or P29) antibody using immunoblotting could be helpful for surgical follow-up.

The disappearance of P40 at 1 year post-surgery was associated with an 87% probability of being relapse-free, and its persistence at the same date was associated with an 83% probability of being relapsed. Hidalgo et al. (2016) described a 40 kDa protein specific to *E. granulosus* protoscoleces which may be involved in host immune responses and infertility of cysts. The authors identified the 40 kDa protein as the nuclear DBF2-related kinase. This enzyme has been reported as essential for many parasites, since its exhaustion disrupts cytokinesis and leads to cell cycle deregulation and cell death [39, 40]. Despite an extensive literature search, no study was found that had tackled the implication of P40 in the post-surgical follow-up of CE. The combination of P40 and doublet P26/27 in this context could be a good strategy, since it enabled the follow-up of 90% of pediatric patients enrolled in this study with an 84% probability of being relapse-free if P40 and/or P26/27 disappeared at 1 year post-surgery, and a 93% probability of being relapsed if P40 and/or P26/27 remained reactive at the same date.

Two other immunoreactive bands of 30 and 46 kDa were identified in 64% and 50% of RCE patients, respectively, and 65% of NRCE patients, and were not useful in predicting relapses as compared to P40 and doublet P26/27. A 30 kDa protein was previously described as specific to *E. granulosus* and identified as prohibitin protein [41]. Li and colleagues (2012) identified a protein of 46 kDa specific to *E. granulosus* protoscolex as myosin, a cell skeleton protein [42]. The P20, the least frequent immunoreactive protein in our survey, does not seem to be useful for the prediction of CE post-surgical outcomes. A previous proteomic study of *E. granulosus* protoscolex antigens reported a protein of 22 kDa as putative peroxiredoxin which may correspond to the immunoreactive band of 20 kDa [42]. It has been previously proven that peroxiredoxin is expressed in *E. granulosus* protoscolex tissue and brood capsules and has DNA protection assay activity [43, 44].

## Conclusions

The B2t recombinant protein appeared to be useful for the prediction of early surgical treatment efficacy, with an 81% probability of being relapse-free when a decrease in antibody level occurs between 1 month and 1 year post-surgery. Furthermore, this valuable information could only be concluded on the basis of a comparative approach between two time points of the monitoring period for each patient due to the individual heterogeneity of immune responses. This comparative approach also appeared to be applicable for evaluating the presence and intensity of immunoblotting bands, especially for the P26/27 and P40. The identified protein bands could serve as novel candidates for the monitoring of CE. In light of these findings, we will focus our further studies on the molecular characterization of these immunoreactive bands. This strategy will hopefully pave the way for identifying novel biomarkers for better management of CE and provide new insights regarding the physiological functions and potential involvement of these proteins in the immunopathogenesis of hydatid cyst disease.

## Data Availability

All data generated or analyzed during this study are included in this published article.

## Abbreviations

CE: Cystic echinococcosis
*E. granulosus*: *Echinococcus granulosus*
HF: Hydatid fluid
IgG: Immunoglobulin G
AgB: Antigen B
RCE: Relapsed cystic echinococcosis patients
NRCE: Non-relapsed cystic echinococcosis patients
SI: Serological index
PSMA: Protoscoleces soluble and membrane antigens of *E. granulosus*
WB: Western blot
CV: Coefficient of variation
IQR: Interquartile range
MW: Molecular weight
